# Variation in Snow Algae Blooms in the Coast Range of British Columbia

**DOI:** 10.3389/fmicb.2020.00569

**Published:** 2020-04-15

**Authors:** Casey B. Engstrom, Kurt M. Yakimovich, Lynne M. Quarmby

**Affiliations:** Department of Molecular Biology and Biochemistry, Simon Fraser University, Burnaby, BC, Canada

**Keywords:** snow, algae, microbiome, amplicon, *rbcL*, 18S, alpine, metabarcoding

## Abstract

Snow algae blooms cover vast areas of summer snowfields worldwide, reducing albedo and increasing snow melt. Despite their global prevalence, little is known about the algae species that comprise these blooms. We used 18S and *rbcL* metabarcoding and light microscopy to characterize algae species composition in 31 snow algae blooms in the Coast Range of British Columbia, Canada. This study is the first to thoroughly document regional variation between blooms. We found all blooms were dominated by the genera *Sanguina, Chloromonas*, and *Chlainomonas*. There was considerable variation between blooms, most notably species assemblages above treeline were distinct from forested sites. In contrast to previous studies, the snow algae genus *Chlainomonas* was abundant and widespread in snow algae blooms. We found few taxa using traditional 18S metabarcoding, but the high taxonomic resolution of *rbcL* revealed substantial diversity, including OTUs that likely represent unnamed species of snow algae. These three cross-referenced datasets (*rbcL*, 18S, and microscopy) reveal that alpine snow algae blooms are more diverse than previously thought, with different species of algae dominating different elevations.

## 1. Introduction

Each summer, vast areas of snow surface are colored red by snow algae blooms in polar and alpine snowfields worldwide. Red snowfields have been found on every continent (Marchant, 1982; Yoshimura et al., 1997; Duval et al., 1999; Segawa et al., 2018; Vimercati et al., 2019) as well as overlying Arctic sea ice (Gradinger and Nurnberg, 1996). Snow algae blooms can be quite extensive: in Alaska, remote sensing suggests snow algae covered up to one third of a 1,900 km^2^ icefield (Ganey et al., 2017). In recent years snow algae have received attention for their role in reducing snow surface albedo, which could substantially increase snow melt (Lutz et al., 2016; Ganey et al., 2017). Thus, snow algae could impact summer water supplies held in mountain snowpack, and reduce glacier mass balance. Snow algae blooms have been recorded throughout history since the time of the ancient Romans (Darwin, 1839; Elder, 1893), but we do not know whether the extent and duration of blooms are increasing with extended melt seasons due to global warming. Despite their potential impact on global albedo, we are only beginning to identify the algae species that comprise snow algae blooms.

Microscopy reveals a diversity of cell morphologies in snow algae blooms, but different species can look nearly identical, and the same species can look completely different depending on environmental conditions (Matsuzaki et al., 2019). The snow algae *Chloromonas krienitzii* are small green biflagellates in culture, but cells in field samples are nearly twice the diameter, with orange pigment, short spines, and thick cell walls (Matsuzaki et al., 2015). The environmental cues that trigger this transformation are not well understood, but increased light intensity and nitrogen deprivation can trigger secondary pigment accumulation in snow algae (Leya et al., 2009), and also in the freshwater algae *Haematococcus pluvialis* (Shah et al., 2016). Green blooms of snow algae are less frequently described in the literature than red blooms, and some researchers have suggested that green snow develops into red snow (Mueller et al., 2001). Metabarcoding studies have found green and red snow with distinct community compositions (Lutz et al., 2015; Terashima et al., 2017), but there are some OTUs that are found in both green and red snow (Lutz et al., 2017), leaving open the possibility that red snow develops from green beginnings.

Green algae of class Chlorophyceae are predominant in many snow algae blooms, including the genera *Sanguina, Chloromonas*, and *Chlainomonas*. The genus *Sanguina* was only recently established and contains just two species; however, many sequences from red snow form a yet-unnamed sister clade to *Sanguina* (Procházková et al., 2019). *Sanguina* has been found in red and orange snow algae blooms worldwide (Procházková et al., 2019). Many species of snow algae have been assigned to *Chloromonas*, including at least twelve cultured representatives (Matsuzaki et al., 2019). Various *Chloromonas* species can form green, orange, or brown colored blooms on the snow surface, and are also found worldwide (Remias et al., 2013, 2018; Procházková et al., 2018b). Less is known about *Chlainomonas*, which has been found in central Europe, western USA, and New Zealand (Novis et al., 2008; Remias et al., 2016; Procházková et al., 2018a). The distinctive red-pigmented cells of this genus (nearly twice the diameter of *Sanguina nivaloides*) have only been reported from waterlogged snow overlying alpine lakes (Novis et al., 2008; Remias et al., 2016; Procházková et al., 2018b). 18S rDNA and *rbcL* sequences show that *Chlainomonas* is closely related to *Chloromonas* (Novis et al., 2008). While Chlorophyceae predominate in many snow algae blooms, other classes of snow algae have been reported: Chrysophyceae in yellow snow in Antarctica, the Alps, and Svalbard (Remias et al., 2019; Soto et al., 2020), and Trebouxiophyceae in green snow in Greenland (Lutz et al., 2015).

While many species of snow algae have been described on the basis of morphology and Sanger sequencing, metabarcoding studies have found red snow algae blooms are dominated by relatively few OTUs. Algae community composition was similar in 33 Arctic red snow samples, all of which were dominated by two OTUs of uncultured Chlamydomonadaceae, along with low relative abundance of *Raphidonema nivale* and *Chloromonas polyptera* (Lutz et al., 2016). Another study using ITS2 metabarcoding found 24 polar red snow sites contained similar algae assemblages, also dominated by two OTUs of uncultured Chlamydomonadaceae with secondary abundance of *Raphidonema* and *Chloromonadinia* (Segawa et al., 2018). Other studies using 18S metabarcoding were limited to class level taxonomic assignments of algae (Hamilton and Havig, 2017)—being highly conserved, short 18S reads cannot distinguish between closely related species or genera.

Based on how little is known about the regional variation in species composition of snow algae blooms, we set out to answer the following questions: what species of snow algae are found in our region? What patterns of co-occurrence exist between species? Which species are the most abundant? Are there distinct bloom types dominated by different species? To answer these questions, we assessed snow algae species composition in 33 samples from the Coast Range of British Columbia using light microscopy and 18S and *rbcL* (coding for Rubisco large subunit) metabarcoding. *rbcL* OTU richness was greater than 18S, revealing previously unknown diversity. By cross-referencing *rbcL*, 18S, and microscopy-based community composition we were able to account for some of the biases inherent in morphology-based identification and PCR-based metabarcoding. Our results show that snow algae species composition was highly variable from site to site, and blooms were dominated by different species at different elevations.

## 2. Materials and Methods

### 2.1. Field Sampling and Microscopy

We collected 309 snow algae samples from alpine and subalpine sites in the Coast Range near Vancouver, British Columbia, Canada throughout the summer of 2018 (Supplementary Figure S1). We collected red, orange, and green snow samples from 13 different mountains from elevations ranging from 880 to 2,150 m above sea level (Supplementary Table S2). We collected samples from progressively higher elevations throughout the season as snow melted at lower elevations. We scooped samples from visibly colored snow into sterile 50 mL tubes, and kept samples cold during transport back to the lab by storing in snow. We melted each sample at room temperature on the lab bench, removed a 1 mL aliquot for light microscopy, and then stored the remaining sample at −20°C for up to eight months until DNA extraction.

We immediately fixed microscopy aliquots in 2% gluteraldehyde, which we stored at 4°C for up to 72 h. We quantified the relative abundance of morphospecies in 122 samples by identifying 100 cells on a haemocytometer under 400x light microscopy. We classified cell morphology based on similarity to published photographs of *Sanguina nivaloides* (Procházková et al., 2019), *Chloromonas* cf. *nivalis* (Procházková et al., 2018b), *Chloromonas* cf. *brevispina* (Matsuzaki et al., 2015), *Chlainomonas krienitzii* (Matsuzaki et al., 2015), *Chlainomonas rubra* (Novis et al., 2008). Cells that did not fall into one of these categories we classified as either “green cell” or “other.” We did not attempt to identify green cells, as different taxa can look highly similar and are therefore prone to misidentification by light microscopy.

### 2.2. DNA Extraction and Amplicon Library Preparation

We selected 33 samples for *rbcL* and 18S metabarcoding. We chose this subset to include samples from different mountains, elevations, and dates, including samples containing distinct or unfamiliar cell morphologies. To lyse the cells we freeze-dried samples and mini-pestled 5–20 mg at room temperature before incubation in CTAB lysis buffer (CTAB extraction buffer, 2009). We extracted DNA in small batches of 5–6 samples at a time using chloroform:isoamyl alcohol (Cubero et al., 1999), and purified DNA using ethanol and spin columns (Qiagen, Hilden) (Supplementary Protocol S3). As a negative control against cross-contamination we processed a tube of sterile distilled water alongside each batch, and tested this for DNA with a Qubit fluorometer (Thermo Fisher, Waltham, MA).

We designed custom primers to target a hypervariable region of snow algae *rbcL*. This gene is an established barcode for green algae, and is highly differentiated at the species level (Hall et al., 2010). We designed primers with the Eurofins primer design tool (https://eurofinsgenomics.eu/en/ecom/tools/pcr-primer-design/) based on the consensus of 20 GenBank snow algae sequences, targeting a 400 bp section of *rbcL* (Supplementary Figure S5). *Sanguina* sequences were not included because they were not available at the time. For 18S we used the universal primers Euk1181 and Euk1624 targeting the V7-V8 hypervariable regions (Wang et al., 2014) (Supplementary Figure S4). Primer sequences are available in Supplementary Figure S6.

We constructed our 18S and *rbcL* amplicon libraries using a standard two-step PCR protocol (Supplementary Protocol S7). The two-step PCR consists of an initial amplification of the region of interest, followed by a secondary amplification that attaches a barcode marker to each oligonucleotide, allowing samples to be pooled for high-throughput sequencing (Meyer and Kircher, 2010). We purified PCR product using Agencourt AMPure XP kit (Beckman Coulter, Brea, CA). We then standardized DNA concentration with Qubit, pooled samples, and sequenced our library on an Illumina MiSeq platform using the V3 kit (Illumina, San Diego, CA).

### 2.3. Bioinformatic Processing

We demultiplexed reads with CUTADAPT (Martin, 2011), and followed the default pipeline of DADA2 to filter, trim, denoise, dereplicate, merge paired-end reads, and remove chimeras (Callahan et al., 2016). We assigned taxonomy to amplicon sequence variants (ASVs) using IDTaxa, discarding assignments with a confidence score of 50% or lower. This is within IDTaxa's recommended settings of 40–60% (Murali et al., 2018). Because snow algae are not well represented on databases such as SILVA, we made custom databases for both 18S and *rbcL* using all available snow algae sequences on GenBank. Additionally, we classified 18S ASV taxonomy with SILVA, using the same 50% confidence cutoff as before (Quast et al., 2013).

We visualized *rbcL* ASV genetic distance using t-SNE (van der Maaten and Hinton, 2008), and then clustered ASVs by sequence similarity into OTUs using DBSCAN (Hahsler et al., 2019) with the epsilon parameter set equal to 4. The output of t-SNE depends on a user-specified parameter “perplexity,” which determines whether the algorithm pays more attention to local or global clustering patterns (Wattenberg et al., 2016). To ensure that our results were not an artifact of this parameter selection, we ran the algorithm with a range of values from 10 to 50 and found no effect on the results. To validate this unconventional OTU clustering method (t-SNE and DBSCAN) we overlaid these OTU clusters on phylogenetic trees using IQTree (Nguyen et al., 2015) (Supplementary Figures S8, S9).

All scripts used in this analysis are freely available at https://github.com/cengstro/bc_snow_algae_amplicon.

## 3. Results

**Figure 1:**
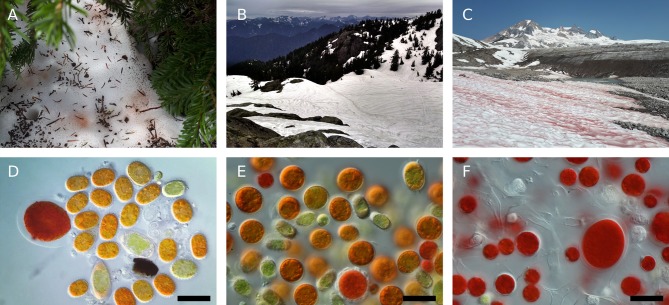
Representative photographs of snow algae in the Coast Mountains of British Columbia. **(A)** Bronze colored snow algae blooms below conifer canopy. **(B)** Dark snow runnels containing snow algae (samples S2.2 and S9.2, Supplementary Table S2). **(C)** Red snow bloom above treeline at sample site G1.1. **(D)** Photomicrograph of bronze snow containing *Chloromonas cf. brevispina* and *Chloromonas cf. nivalis*. All photomicrographs taken with DIC light microscopy at 630x magnification, all scale bars 30 μm. **(E)** Photomicrograph of orange snow from the surface of a runnel containing *Chloromonas krienitzii* and *Chloromonas cf. nivalis*. **(F)** Photomicrograph of red snow containing *Sanguina nivaloides* and *Chlainomonas rubra* cell morphologies.

We observed morphologically distinct snow algae blooms at different elevations (Figure 1; Supplementary Figure S10). We observed green, orange, and red snow as early as May 18 in forested areas, but did not observe snow algae above treeline (approximately 1,500 m in our study region) until June 20 (Supplementary Table S2). Red snow was prevalent in areas of high solar exposure above treeline, and most of these sites were dominated by cell morphologies we classified as *Sanguina cf. nivaloides*. Cells resembling *Chlainomonas rubra* were common at all elevations, often as the dominant cell type. Below treeline, the dominant cell morphologies were classified as *Chloromonas cf. brevispina, Chloromonas cf. nivalis*, and green cells that we did not attempt to classify.

**Figure 2:**
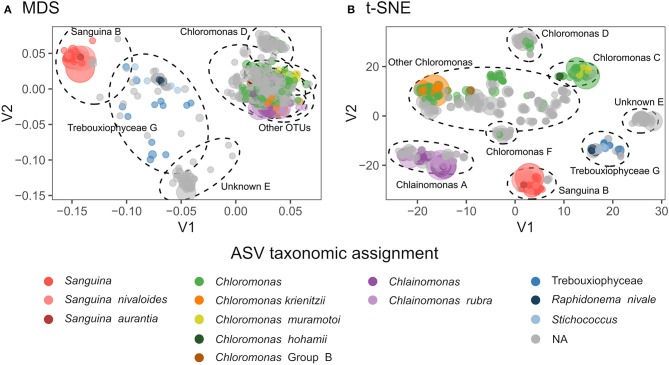
**(A)** Multidimensional scaling (MDS) plot showing genetic distances between *rbcL* ASVs. Taxonomic assignments are indicated by color, and point size is proportional to total relative abundance. Dotted ellipses indicate OTU clusters. Stress = 0.13. **(B)** t-Distributed Stochastic Neighbor Embedding (t-SNE) dimensionality reduction of snow algae *rbcL* ASVs, with perplexity = 30. Dotted lines indicate OTU clustering by DBSCAN with eps=4.

Both 18S and *rbcL* amplicon libraries were dominated by reads assigned to Chlorophyta (Figure 2). We detected 68 algae amplicon sequence variants (ASVs) using 18S: 50 Chlorophyceae, 11 Trebouxiophyceae, and 7 Chrysophyceae. Our *rbcL* library detected 644 ASVs: 603 Chlorophyta and 41 Trebouxiophyceae. We found seven distinct *rbcL* ASV clusters (which we define here as OTUs) compared with just three 18S-defined algal OTUs (Supplementary Figure S9). In *rbcL*, the most abundant genera were *Chloromonas, Chlainomonas*, and *Sanguina*. Although the majority of ASVs were not assigned to genus level, our clustering showed that most ASVs were genetically similar to one of these three genera (Figure 2). OTUs “D” and “F” were closely related to *Chloromonas*, but they did not match any known species on GenBank. OTU “E” was not assigned to genus level, and the ten best BLAST matches included six different genera within Chlamydomonadaceae (86–87% sequence match), two of which were *Chloromonas* snow algae (LC012735).

**Figure 3:**
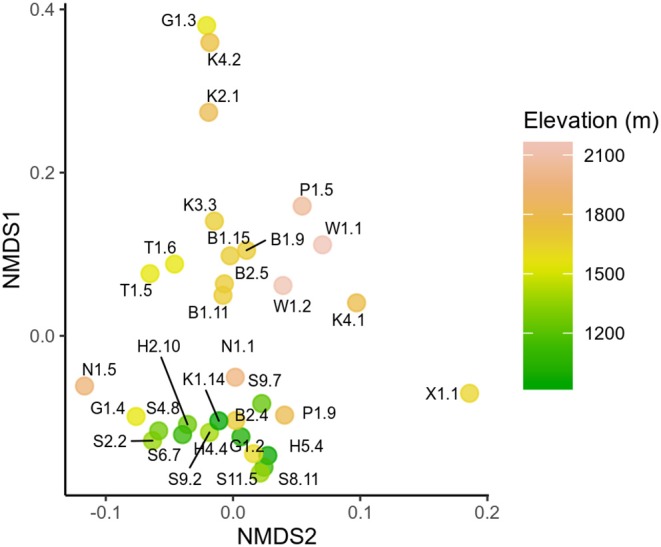
Non-metric multidimensional scaling (NMDS) showing *rbcL* UniFrac distances between samples. Each sample is labeled by sample ID and colored by elevation.

**Figure 4:**
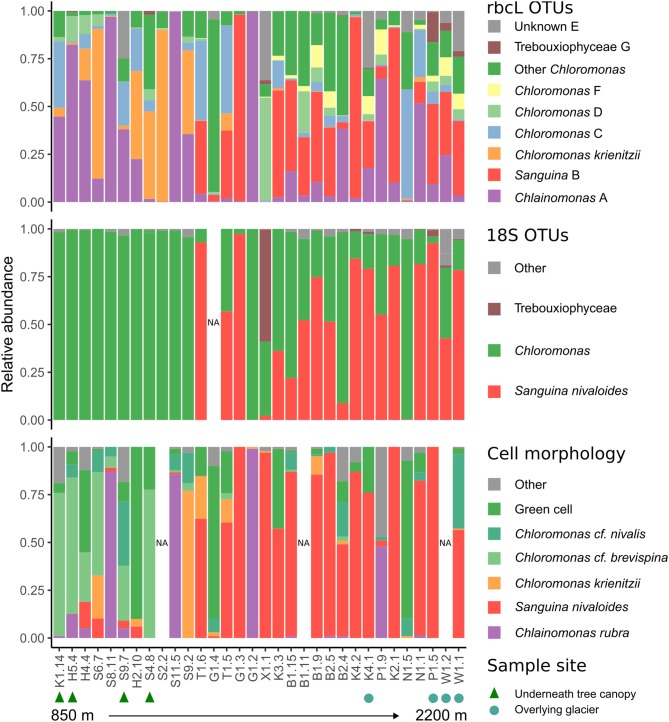
Stacked barplots showing snow algae relative abundance, as measured by: **(top)** *rbcL* OTUs, **(middle)** 18S taxonomic assignment with custom snow algae database, and **(bottom)** cell morphology. Samples are arranged from low to high elevation left to right. Green triangles **(bottom)** indicate samples collected from sites below conifer canopy, and blue squares indicate samples collected from snow overlying glacier.

18S and *rbcL* taxonomic composition varied with elevation. Low elevation samples were similar as shown by low *rbcL* UniFrac distances (Lozupone and Knight, 2005), while generally high elevation samples were more compositionally distinct (Figure 3). Samples collected latest in the season had the highest diversity (Supplementary Figure S11). Although there was no statistically significant trend between Shannon diversity and date, there was a weak correlation between Faith's phylogenetic diversity and date (Pearson's *r* = 0.36, *p* = 0.04). *Sanguina* predominated above 1,500 m, but was absent below this elevation (Figure 4). High-elevation samples contained one OTU of *Chloromonas* that was absent from low-elevation sites (Figure 3 OTU “F”). *Raphidonema* was restricted to three samples from high-elevation snow overlying glaciers (best BLAST match to *Raphidonema longiseta*, KM462868.1). Most snow algae blooms above treeline were red, but we did collect two green snow samples from above treeline (N1.5, G1.4), both of which were dominated by *Chloromonas*. *Chlainomonas* was highly abundant at all elevations, in both high and low relative abundance. *Chloromonas krienitzii* was predominant around 1,200 m in clearings or sparse trees.

At one site, we observed the snow transition from green to orange (Supplementary Figure S12). In May, the surface snow was white, with green snow 2–5 cm below the surface of a runnel (sample S2.2). On subsequent visits in June, the same runnel was orange on the surface with the green snow remaining below the surface (samples S4.6, S6.1, S9.2, S11.2). The green snow contained predominantly green cells, including cells with 2 or 4 flagella, while the orange snow contained predominantly orange spherical cells resembling *Chloromonas krienitzii* (Matsuzaki et al., 2015). Both green and orange snow were dominated by *rbcL* reads assigned to *C. krienitzii*, although the orange surface snow contained slightly higher abundance of *Chlainomonas* (Supplementary Figure S12).

## 4. Discussion

Snow algae blooms are a widespread and globally important phenomenon, yet until now the distribution of distinct blooms within a region has not been well-documented. We present multiple data sets demonstrating elevational patterns in alpine snow algae bloom species composition. Most dramatically, *Sanguina* was dominant in red snow above treeline, while green and orange blooms of *C. krienitzii* were dominant in runnels at lower elevations. We found unexpected diversity within *rbcL* that we did not detect using 18S, including yet-unnamed species.

Community composition was consistent between 18S and *rbcL* libraries. However, in some cases we were able to distinguish taxa by *rbcL* that were not distinguished with 18S. For example, *Chlainomonas* was absent from our 18S dataset (Figure 4), likely because 18S of this genus is nearly identical to that of the closely-related *Chloromonas*. Because of this similarity, previous metabarcoding studies using 18S may have missed this genus. Indeed, our *rbcL* data suggest *Chlainomonas* may be more widely distributed than previously thought. Although previous work suggested that *Chlainomonas* is restricted to waterlogged snow overlying lakes (Novis et al., 2008; Procházková et al., 2018a), we did not find this to be the case. Only one *Chlainomonas*-dominant sample was located in waterlogged snow at the edge of a melt pool (sample S8.11), while the other *Chlainomonas*-dominant sample sites were not notably wetter than the surrounding snow, nor were they located over frozen lakes.

We noted that *Chlainomonas* was found in consistently higher relative abundance in our *rbcL* dataset than in cell counts (Figure 3). One possible explanation is that *rbcL* could have overestimated *Chlainomonas* due to higher *rbcL* copy number. *rbcL* is located in the plastid genome, and *Chlainomonas rubra* has multiple parietal chloroplasts per cell (Procházková et al., 2018b). Thus, *Chlainomonas* could have more copies of the plastid genome than genera with only one chloroplast such as *Sanguina* (Procházková et al., 2019). However, *Chlamydomonas reinhardtii* plastid genome copy number can vary depending on growth conditions (Eberhard et al., 2002). Accounting for the discrepancy between cell counts and rbcL relative abundance could prove challenging.

Our findings highlight the remaining unexplored diversity in the snow algae microbiome. Many ASVs were closely related to *Chloromonas*. Two *Chloromonas* OTUs “D” and “F” did not match any known species in GenBank (Figure 2). The majority of *Chloromonas rbcL* ASVs did not form distinct clusters. There were at least three species present in this group, however there could be more. One possibility is the 18S of OTU “E” is identical to other algae 18S. Indeed, *rbcL* diversity could be higher within *Chloromonas* than other genera because most *Chloromonas* species lack a pyrenoid (Nozaki et al., 2002), an organelle involved in the carbon-concentrating mechanism which contains high concentrations of cross-linked Rubisco. Perhaps due to the absence of a pyrenoid, many species of *Chloromonas* have high concentrations of non-synonymous mutations in the region of *rbcL* that codes for binding Rubisco together (Nozaki et al., 2002). While *rbcL* is a poor indicator of *Chloromonas* phylogeny (Nozaki et al., 2002), it nonetheless is highly differentiated between species and therefore is an effective barcode (Hall et al., 2010). Future studies could use ITS2, because compensatory base changes (CBCs) in this region correlate with species boundaries (Wolf et al., 2013).

The variation in species composition we observed could be due to differences in environmental conditions. Notably, *Sanguina* and *Chloromonas* “C” were only found in late summer samples from high alpine sites above 1,500 m (Figure 3). The lack of canopy cover at high elevations could select for light-tolerant species, but many low-elevation blooms were found in clearings that also received full sunlight (Figure 4). Nutrients could also impact snow algae species composition: garden fertilizer applied to experimental plots in Alaska stimulated snow algae growth (Ganey et al., 2017), and conifer litter was found to enhance the growth of the cultures of *Chloromonas pichinchae*, but not *Raphidonema nivale* (Hoham, 1976). Intriguingly, *Chloromonas* was dominant in low elevation sites with canopy cover, while we only found *Raphidonema* at high-elevation glacier sites. In Svalbard, *R. nivale* abundance increased on glacier surface snow following wind storms, and the authors concluded that *Raphidonema* is a soil algae that can opportunistically colonize snow via wind (Stibal and Elster, 2005). Given the aerial dispersal capabilities of microalgae (Tesson et al., 2016) and genetic overlap between Arctic and Antarctic snow algae populations (Segawa et al., 2018) it seems unlikely that geographic distance would pose a barrier to snow algae distribution on a regional scale. Day length could also explain some of the seasonal variation we observed: peak snowmelt would coincide with longer day length at our high elevation sites, whereas snowmelt coincides with shorter days at lower elevations. Site topography could also potentially influence species distribution: two sites dominated by *C. krienitzii* were in runnels overlying ephemeral streams, which could influence snow moisture and nutrient availability. However, measuring these environmental parameters is more challenging than it might initially appear. For example, depending on the time of day and weather we could visit the same bloom and get completely different measurements of temperature, irradiance, or snow moisture. The need for environmental data preceding the blooms is one of several issues that will be addressed in future work.

Previous work has shown that *Chloromonas krienitzii* undergoes distinct green and orange morphologies (Matsuzaki et al., 2015), but our study is the first to document this transition in the field. The transition occurred from May to June, suggesting that this process is mediated by seasonal changes. Although *Chlainomonas rbcL* was abundant in orange surface samples, our microscopic observations suggest that this was not the species responsible for the orange coloration, as orange *C. krienitzii* far outnumbered the red *Chlainomonas*. Secondary pigments likely protect snow algae from the damaging effects of intense solar irradiation at the snow surface (Bidigare et al., 1993), which could be why green cells were concentrated a few centimeters below the snow surface.

In conclusion, snow algae blooms contain diverse species assemblages, with different species occurring at different elevations. Blooms were dominated by three genera, *Chloromonas, Chlainomonas*, and *Sanguina*. We report substantially more species-level diversity than previous studies based on morphology or 18S sequence. Our work provides insight into the diversity and distribution of snow algae, the primary producers in a poorly understood yet globally important microbiome.

## Data Availability Statement

All raw sequence data are available under European Nucleotide Archive accession PRJEB34539. All scripts used in this study are available at https://github.com/cengstro/bc_snow_algae_amplicon.

## Author Contributions

CE, LQ, and KY designed this study. Samples collected by CE with assistance from KY and LQ. KY and CE prepared sequence libraries. CE and KY completed the bioinformatic analysis. CE wrote the manuscript with major input from LQ and KY. All authors discussed the results and contributed to the final manuscript.

### Conflict of Interest

The authors declare that the research was conducted in the absence of any commercial or financial relationships that could be construed as a potential conflict of interest.

## References

[B1] Bidigare R. R. Ondrusek M. E. Kennicutt M. C. Iturriaga R. Harvey H. R. Hoham R. W. (1993). Evidence a photoprotective for secondary carotenoids of snow algae. J. Phycol. 29, 427–434. 10.1111/j.1529-8817.1993.tb00143.x

[B2] Callahan B. J. McMurdie P. J. Rosen M. J. Han A. W. Johnson A. J. A. Holmes S. P. (2016). DADA2: High-resolution sample inference from Illumina amplicon data. Nat. Methods 13, 581–583. 10.1038/nmeth.3869 27214047 PMC4927377

[B3] CTAB extraction buffer (2009). Cold Spring Harbor Protocols 2009 10.1101/pdb.rec11984

[B4] Cubero O. F. Crespo A. Fatehi J. Bridge P. D. (1999). DNA extraction and PCR amplification method suitable for fresh, herbarium-stored, lichenized, and other fungi. Plant Syst. Evol. 216, 243–249. 10.1007/BF01084401

[B5] Darwin C. (1839). The Voyage of the Beagle. London; New York, NY: Dent; Dutton. Available online at: https://search.library.wisc.edu/catalog/999467080402121

[B6] Duval B. Duval E. Hoham R. W. (1999). Snow algae of the Sierra Nevada, Spain, and High Atlas mountains of Morocco. Int. Microbiol. 2, 39–42. 10943390

[B7] Eberhard S. Drapier D. Wollman F.-A. (2002). Searching limiting steps in the expression of chloroplast-encoded proteins: relations between gene copy number, transcription, transcript abundance and translation rate in the chloroplast of *Chlamydomonas reinhardtii*. Plant J. 31, 149–160. 10.1046/j.1365-313X.2002.01340.x 12121445

[B8] Elder P. (1893). “Chap. 57 (56.) Showers of milk, blood, flesh, iron, wool, and baked tiles” in The Natural History of Pliny, Vol. 1 (London: George Bell & Sons). Available online at: https://books.google.ca/books?id=MZYVAAAAYAAJ&printsec=frontcover&dq=Showers+of+milk,+blood,+flesh,+iron,+wool,+and+baked+tiles%27%27+in+The+Natural+History+of+Pliny,+Volume+1.+Bohn%27s+Classical+Library&hl=en&sa=X&ved=0ahUKEwiB_sqO1cXoAhUkJDQIHR7KA8QQ6AEIKDAA#v=onepage&q&f=false

[B9] Ganey G. Q. Loso M. G. Burgess A. B. Dial R. J. (2017). The role of microbes in snowmelt and radiative forcing on an Alaskan icefield. Nat. Geosci. 10, 754–759. 10.1038/ngeo3027

[B10] Gradinger R. Nurnberg D. (1996). Snow algal communities on arctic pack ice floes dominated by *Chlamydomonas nivalis* (Bauer) Wille. Proc. NIPR Symp. Polar Biol. 9, 35–43.

[B11] Hahsler M. Piekenbrock M. Doran D. (2019). Dbscan: fast density-based clustering with R. J. Stat. Softw. 91, 1–29. 10.18637/jss.v091.i01

[B12] Hall J. D. Fu K. Lo C. Lewis L. A. Karol K. G. (2010). An assessment of proposed DNA barcodes in freshwater green algae. Cryptog. Algol. 31, 529–555.

[B13] Hamilton T. L. Havig J. (2017). Primary productivity of snow algae communities on stratovolcanoes of the Pacific Northwest. Geobiology 15, 280–295. 10.1111/gbi.12219 27917584 PMC5324535

[B14] Hoham R. W. (1976). The effect of coniferous litter and different snow meltwaters upon the growth of two species of snow algae in axenic culture. Arct. Alpine Res. 8, 377–386. 10.1080/00040851.1976.12003886

[B15] Leya T. Rahn A. Lütz C. Remias D. (2009). Response of arctic snow and permafrost algae to high light and nitrogen stress by changes in pigment composition and applied aspects for biotechnology: pigment change in snow algae. FEMS Microbiol. Ecol. 67, 432–443. 10.1111/j.1574-6941.2008.00641.x 19159422

[B16] Lozupone C. Knight R. (2005). UniFrac: a new phylogenetic method for comparing microbial communities. Appl. Environ. Microbiol. 71, 8228–8235. 10.1128/AEM.71.12.8228-8235.2005 16332807 PMC1317376

[B17] Lutz S. Anesio A. M. Edwards A. Benning L. G. (2017). Linking microbial diversity and functionality of arctic glacial surface habitats. Environ. Microbiol. 19, 551–565. 10.1111/1462-2920.13494 27511455

[B18] Lutz S. Anesio A. M. Field K. Benning L. G. (2015). Integrated “Omics”, targeted metabolite and single-cell analyses of arctic snow algae functionality and adaptability. Front. Microbiol. 6:1323. 10.3389/fmicb.2015.01323 26635781 PMC4659291

[B19] Lutz S. Anesio A. M. Raiswell R. Edwards A. Newton R. J. Gill F.. (2016). The biogeography of red snow microbiomes and their role in melting arctic glaciers. Nat. Commun. 7:11968. 10.1038/ncomms11968 27329445 PMC4917964

[B20] Marchant H. J. (1982). Snow algae from the Australian snowy mountains. Phycologia 21, 178–184. 10.2216/i0031-8884-21-2-178.1

[B21] Martin M. (2011). Cutadapt removes adapter sequences from high-throughput sequencing reads. EMBnet J. 17, 10–12. 10.14806/ej.17.1.200

[B22] Matsuzaki R. Kawai-Toyooka H. Hara Y. Nozaki H. (2015). Revisiting the taxonomic significance of aplanozygote morphologies of two cosmopolitan snow species of the genus Chloromonas (Volvocales, Chlorophyceae). Phycologia 54, 491–502. 10.2216/15-33.1

[B23] Matsuzaki R. Nozaki H. Takeuchi N. Hara Y. Kawachi M. (2019). Taxonomic re-examination of “Chloromonas nivalis (Volvocales, Chlorophyceae) zygotes” from Japan and description of *C. muramotoi sp. nov*. PLoS ONE 14:e0210986 10.1371/journal.pone.0210986 30677063 PMC6345437

[B24] Meyer M. Kircher M. (2010). Illumina sequencing library preparation for highly multiplexed target capture and sequencing. Cold Spring Harb. Protoc. 2010:pdb.prot5448. 10.1101/pdb.prot5448 20516186

[B25] Mueller T. Leya T. Fuhr G. (2001). Persistent snow algal fields in spitsbergen: field observations and a hypothesis about the annual cell circulation. Arctic Antarct. Alpine Res. 33, 42–51. 10.2307/1552276

[B26] Murali A. Bhargava A. Wright E. S. (2018). IDTAXA: A novel approach for accurate taxonomic classification of microbiome sequences. Microbiome 6:140. 10.1186/s40168-018-0521-5 30092815 PMC6085705

[B27] Nguyen L.-T. Schmidt H. A. Haeseler A. von, Minh, B. Q. (2015). IQ-TREE: a fast and effective stochastic algorithm for estimating maximum-likelihood phylogenies. Mol. Biol. Evol. 32, 268–274. 10.1093/molbev/msu300 25371430 PMC4271533

[B28] Novis P. M. Hoham R. W. Beer T. Dawson M. (2008). Two snow species of the quadriflagellate green alga Chlainomonas (Chlorophyta, Volvocales): ultrastructure and phylogenetic position within the Chloromonas clade. J. Phycol. 44, 1001–1012. 10.1111/j.1529-8817.2008.00545.x 27041619

[B29] Nozaki H. Onishi K. Morita E. (2002). Differences in pyrenoid morphology are correlated with differences in the rbcL genes of members of the chloromonas lineage (Volvocales, Chlorophyceae). J. Mol. Evol. 55, 414–430. 10.1007/s00239-002-2338-9 12355262

[B30] Procházková L. Leya T. Křížková H. Nedbalová L. (2019). *Sanguina nivaloides* and *Sanguina aurantia* gen. Et spp. Nov. (Chlorophyta): the taxonomy, phylogeny, biogeography and ecology of two newly recognised algae causing red and orange snow. FEMS Microbiol. Ecol. 95:fiz064. 10.1093/femsec/fiz064 31074825 PMC6545352

[B31] Procházková L. Remias D. Holzinger A. Řezanka T. Nedbalová L. (2018a). Ecophysiological and morphological comparison of two populations of *Chlainomonas* sp. (Chlorophyta) causing red snow on ice-covered lakes in the High Tatras and Austrian Alps. Eur. J. Phycol. 53, 230–243. 10.1080/09670262.2018.1426789 29755214 PMC5940174

[B32] Procházková L. Remias D. Rezanka T. Nedbalova L. (2018b). Chloromonas nivalis subsp. Tatrae, subsp. Nov. (Chlamydomonadales, Chlorophyta): re-examination of a snow alga from the High Tatra Mountains (Slovakia). Fottea 18, 1–18. 10.5507/fot.2017.010 30976329 PMC6456015

[B33] Quast C. Pruesse E. Yilmaz P. Gerken J. Schweer T. Yarza P.. (2013). The SILVA ribosomal RNA gene database project: improved data processing and web-based tools. Nucleic Acids Res. 41, D590–D596. 10.1093/nar/gks1219 23193283 PMC3531112

[B34] Remias D. Pichrtová M. Pangratz M. Lütz C. Holzinger A. (2016). Ecophysiology, secondary pigments and ultrastructure of *Chlainomonas* sp. (Chlorophyta) from the European Alps compared with *Chlamydomonas nivalis* forming red snow. FEMS Microbiol. Ecol. 92:fiw030. 10.1093/femsec/fiw030 26884467 PMC4815433

[B35] Remias D. Procházková L. Holzinger A. Nedbalová L. (2018). Ecology, cytology and phylogeny of the snow alga *Scotiella cryophila* K-1 (Chlamydomonadales, Chlorophyta) from the Austrian Alps. Phycologia 57, 581–592. 10.2216/18-45.1 31007285 PMC6469580

[B36] Remias D. Procházková L. Nedbalová L. Andersen R. A. Valentin K. (2019). Two new Kremastochrysopsis species, *K. austriaca* sp. Nov. And *K. americana* sp. Nov. (Chrysophyceae). J. Phycol. jpy.12937. 10.1111/jpy.12937 31639884 PMC7054049

[B37] Remias D. Wastian H. Lütz C. Leya T. (2013). Insights into the biology and phylogeny of *Chloromonas polyptera* (Chlorophyta), an alga causing orange snow in Maritime Antarctica. Antarct. Sci. 25, 648–656. 10.1017/S0954102013000060

[B38] Segawa T. Matsuzaki R. Takeuchi N. Akiyoshi A. Navarro F. Sugiyama S.. (2018). Bipolar dispersal of red-snow algae. Nat. Commun. 9:3094. 10.1038/s41467-018-05521-w 30082897 PMC6079020

[B39] Shah M. M. R. Liang Y. Cheng J. J. Daroch M. (2016). Astaxanthin-producing green microalga *Haematococcus pluvialis*: from single cell to high value commercial products. Front. Plant Sci. 7:531. 10.3389/fpls.2016.00531 27200009 PMC4848535

[B40] Soto D. F. Fuentes R. Huovinen P. Gómez I. (2020). Microbial composition and photosynthesis in Antarctic snow algae communities: integrating metabarcoding and pulse amplitude modulation fluorometry. Algal Res. 45:101738 10.1016/j.algal.2019.101738

[B41] Stibal M. Elster J. (2005). Growth and morphology variation as a response to changing environmental factors in two Arctic species of Raphidonema (Trebouxiophyceae) from snow and soil. Polar Biol. 28, 558–567. 10.1007/s00300-004-0709-y

[B42] Terashima M. Umezawa K. Mori S. Kojima H. Fukui M. (2017). Microbial community analysis of colored snow from an alpine snowfield in Northern Japan reveals the prevalence of betaproteobacteria with snow algae. Front. Microbiol. 8:1481. 10.3389/fmicb.2017.01481 28824603 PMC5545588

[B43] Tesson S. V. M. Skjøth C. A. S?^antl-Temkiv T. Löndahl J. (2016). Airborne microalgae: insights, opportunities, and challenges. Appl. Environ. Microbiol. 82, 1978–1991. 10.1128/AEM.03333-15 26801574 PMC4807511

[B44] van der Maaten L. Hinton G. (2008). Visualizing data using t-SNE. J. Mach. Learn. Res. 9, 2579–2605.

[B45] Vimercati L. Solon A. J. Krinsky A. Arán P. Porazinska D. L. Darcy J. L. (2019). Nieves penitentes are a new habitat for snow algae in one of the most extreme high-elevation environments on Earth. Arctic Antarct. Alpine Res. 51, 190–200. 10.1080/15230430.2019.1618115

[B46] Wang Y. Tian R. M. Gao Z. M. Bougouffa S. Qian P.-Y. (2014). Optimal eukaryotic 18S and universal 16S/18S ribosomal RNA primers and their application in a study of symbiosis. PLoS ONE 9:e90053. 10.1371/journal.pone.0090053 24594623 PMC3940700

[B47] Wattenberg M. Viégas F. Johnson I. (2016). How to use t-SNE effectively. Distill 1:e2 10.23915/distill.00002

[B48] Wolf M. Chen S. Song J. Ankenbrand M. Müller T. (2013). Compensatory base changes in ITS2 secondary structures correlate with the biological species concept despite intragenomic variability in ITS2 sequences – a proof of concept. PLoS ONE 8:e66726. 10.1371/journal.pone.0066726 23826120 PMC3691174

[B49] Yoshimura Y. Kohshima S. Ohtani S. (1997). A community of snow algae on a Himalayan glacier: change of algal biomass and community structure with altitude. Arctic Alpine Res. 29:126 10.2307/1551843

