# Supplementary material for: Variation in Snow Algae Blooms in the Coast Range of British Columbia
Source: Front Microbiol. 2020 Apr 15;11:569. doi: 10.3389/fmicb.2020.00569 (PMC7174675; doi:10.3389/fmicb.2020.00569)
Supplement: Supplementary file 1 [file Data_Sheet_1.pdf]

# Supplementary materials

## Contents

**S1. Map of sample locations** **2**

**S2. Table of sample site data** **3**

**S3. DNA extraction protocol** **3**

**S4. Comparison of 18S GenBank sequences** **4**

**S5. Comparison of *rbcL* GenBank sequences** **5**

**S6. *rbcL* and 18S primer sequences** **6**

**S7. PCR conditions used for Illumina amplicon library prep** **6**

**S8. Phylygenetic tree of *rbcL* ASVs** **7**

**S9. 18S Chlorophyceae ASV clustering** **8**

**S10. Cell count relative abundance** **9**

**S11. *rbcL* sample diversity by date and elevation** **10**

**S12. Community comparison: same site, different dates & depths** **11**

## S1. Map of sample locations

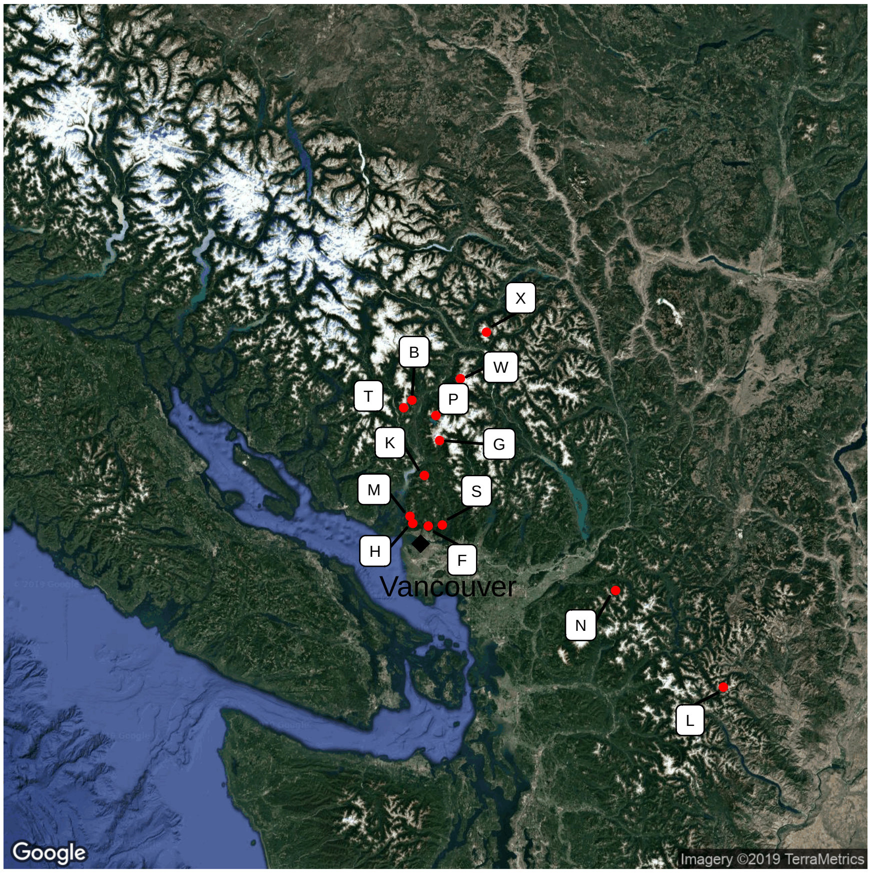


**Supplementary figure S1**. Map showing snow algae sample locations relative to city of Vancouver (black diamond). Letters indicate mountain names: X=Saxifrage, W=Wedge, B=Brew, P=Panorama Ridge, T=Tricouni, K=Sky Pilot, G=Garibaldi, M=St. Marks summit, H=Hollyburn, F=Fromme, S=Seymour, N=Nesakwatch, L=Liberty Bell. Created using the googleway and ggmap packages in R.

## S2. Table of sample site data

**Supplementary table S2.** Site data for samples included in metabarcoding analysis. Sample letter indicates mountain of origin, numbers indicate unique sample identifier.

| Sample ID | Date | Latitude | Longitude | Elevation (m) | Color snow | Habitat |
|---|---|---|---|---|---|---|
| H2.10 | 2018-05-18 | 49.39847 | -123.1840 | 1246.300 | green | clearing |
| K1.14 | 2018-05-20 | 49.65536 | -123.1062 | 945.500 | green | forest |
| S2.2 | 2018-05-22 | 49.38775 | -122.9425 | 1364.400 | green | alpine |
| S4.8 | 2018-06-05 | 49.38361 | -122.9417 | 1257.200 | rusty | forest |
| H4.4 | 2018-06-11 | 49.38358 | -123.1844 | 998.300 | orange | clearing |
| H5.4 | 2018-06-14 | 49.38076 | -123.1875 | 957.600 | pink | forest |
| S6.7 | 2018-06-14 | 49.37437 | -122.9502 | 1089.400 | orange | clearing |
| K2.1 | 2018-06-20 | 49.63519 | -123.0923 | 1804.800 | pink | alpine |
| T1.5 | 2018-06-23 | 50.00485 | -123.2585 | 1550.000 | pink | alpine |
| T1.6 | 2018-06-23 | 50.00547 | -123.2574 | 1549.900 | pink | alpine |
| S8.11 | 2018-06-28 | 49.37532 | -122.9464 | 1143.800 | pink | clearing |
| S9.2 | 2018-07-04 | 49.38769 | -122.9413 | 1382.100 | orange | clearing |
| S9.7 | 2018-07-04 | 49.38006 | -122.9420 | 1222.600 | orange | forest |
| K3.3 | 2018-07-07 | 49.63924 | -123.0907 | 1666.700 | orange | alpine |
| N1.1 | 2018-07-14 | 49.03335 | -121.5330 | 1957.800 | pink | alpine |
| N1.5 | 2018-07-14 | 49.03405 | -121.5338 | 1926.100 | green | alpine |
| S11.5 | 2018-07-19 | 49.38656 | -122.9419 | 1372.000 | orange | alpine |
| B1.9 | 2018-07-21 | 50.03934 | -123.1907 | 1678.500 | pink | alpine |
| B1.11 | 2018-07-21 | 50.03977 | -123.1907 | 1678.300 | pink | alpine |
| B1.15 | 2018-07-21 | 50.03947 | -123.1899 | 1673.300 | pink | alpine |
| B2.4 | 2018-07-22 | 50.03940 | -123.1909 | 1681.500 | orange | alpine |
| B2.5 | 2018-07-22 | 50.03940 | -123.1909 | 1680.900 | other | other |
| G1.2 | 2018-07-27 | 49.82753 | -122.9660 | 1600.000 | pink | alpine |
| G1.3 | 2018-07-27 | 49.82617 | -122.9637 | 1553.600 | pink | alpine |
| G1.4 | 2018-07-27 | 49.82775 | -122.9666 | 1550.000 | green | alpine |
| X1.1 | 2018-07-28 | 50.39219 | -122.5844 | 1629.800 | pink | alpine |
| W1.1 | 2018-08-13 | 50.15007 | -122.7970 | 2136.000 | pink | glacier |
| W1.2 | 2018-08-13 | 50.15028 | -122.7966 | 2125.000 | other | glacier |
| K4.1 | 2018-08-26 | 49.63601 | -123.0936 | 1771.651 | orange | glacier |
| K4.2 | 2018-08-26 | 49.63631 | -123.0912 | 1742.805 | pink | alpine |
| P1.5 | 2018-09-08 | 49.95646 | -123.0098 | 2048.702 | pink | glacier |
| P1.9 | 2018-09-08 | 49.97357 | -123.0031 | 1789.000 | other | other |

## S3. DNA extraction protocol

To extract DNA from the crushed cells we added 800 mL CTAB (Cold Springs Harbour Protocols), 1% B-mercaptoethanol, 5 $\mu$L each of proteinase K and RNAase A, and incubated these at 65 °C for 30 minutes. We spun samples at 10,000 g for 3 minutes to pellet cell debris, then added 700 $\mu$L of supernatant to an equal volume of 24:1 chloroform:isoamyl alcohol. We inverted samples to mix and centrifuged again at 12,000 g for 10 minutes. We precipitated DNA by transferring the top layer to 700 $\mu$L ice-cold ethanol, which we gently mixed, and spun over Qiagen DNA columns for 30 s at 15,000 g. We washed columns twice with 70% ethanol, and finally dissolved our DNA by spinning with 50 $\mu$L of sterile TE buffer.

# S4. Comparison of 18S GenBank sequences

A.

| | 1. *Chlamydomonas nivalis* (GU117577) | 2. Chlainomonas sp. LP03 (MF803745) | 3. *Chloromonas nivalis* tetrae (KY499614.1) | 4. *Chloromonas fukushimae* (AB906342) | 5. *Chloromonas tughillensis* (AB734116) | 6. *Chloromonas nivalis* (GU117576) | 7. *Chloromonas polyptera* (JQ790556) | 8. *Chloromonas hohamii* (AB906344) | 9. *Chloromonas brevispina* (AF517092) | 10. *Chloromonas chenangoensis* (AB734113) | 11. *Raphidonema nivalis* (AJ306532) | 12. *Raphidonema sempervirens* (AF514410) | 13. *Pseuodchlorella signiensis* (KM116465) | 14. *Pseudochlorella pringsheimii* (FM958479) |
|---|---|---|---|---|---|---|---|---|---|---|---|---|---|---|
| *Chlamydomonas nivalis* (GU117577) 1. | 0 | | | | | | | | | | | | | |
| Chlainomonas sp. LP03 (MF803745) 2. | 9 | 0 | | | | | | | | | | | | |
| *Chloromonas nivalis* tetrae (KY499614.1) 3. | 9 | 0 | 0 | | | | | | | | | | | |
| *Chloromonas fukushimae* (AB906342) 4. | 9 | 0 | 0 | 0 | | | | | | | | | | |
| *Chloromonas tughillensis* (AB734116) 5. | 11 | 2 | 2 | 2 | 0 | | | | | | | | | |
| *Chloromonas nivalis* (GU117576) 6. | 9 | 0 | 0 | 0 | 2 | 0 | | | | | | | | |
| *Chloromonas polyptera* (JQ790556) 7. | 12 | 3 | 3 | 3 | 5 | 3 | 0 | | | | | | | |
| *Chloromonas hohamii* (AB906344) 8. | 11 | 2 | 2 | 2 | 0 | 2 | 5 | 0 | | | | | | |
| *Chloromonas brevispina* (AF517092) 9. | 15 | 6 | 6 | 6 | 4 | 6 | 9 | 4 | 0 | | | | | |
| *Chloromonas chenangoensis* (AB734113) 10. | 9 | 0 | 0 | 0 | 2 | 0 | 3 | 2 | 6 | 0 | | | | |
| *Raphidonema nivalis* (AJ306532) 11. | 32 | 31 | 31 | 31 | 31 | 31 | 33 | 31 | 33 | 31 | 0 | | | |
| *Raphidonema sempervirens* (AF514410) 12. | 32 | 31 | 31 | 31 | 31 | 31 | 33 | 31 | 33 | 31 | 0 | 0 | | |
| *Pseuodchlorella signiensis* (KM116465) 13. | 33 | 32 | 32 | 32 | 30 | 32 | 34 | 30 | 32 | 32 | 1 | 1 | 0 | |
| *Pseudochlorella pringsheimii* (FM958479) 14. | 30 | 30 | 30 | 30 | 30 | 30 | 32 | 30 | 32 | 30 | 2 | 2 | 3 | 0 |

B.

| | 1. *Chlamydomonas nivalis* (GU117577) | 2. Chlainomonas sp. LP03 (MF803745) | 3. *Chloromonas nivalis* tetrae (KY499614.1) | 4. *Chloromonas fukushimae* (AB906342) | 5. *Chloromonas tughillensis* (AB734116) | 6. *Chloromonas nivalis* (GU117576) | 7. *Chloromonas polyptera* (JQ790556) | 8. *Chloromonas hohamii* (AB906344) | 9. *Chloromonas brevispina* (AF517092) | 10. *Chloromonas chenangoensis* (AB734113) | 11. *Raphidonema nivalis* (AJ306532) | 12. *Raphidonema sempervirens* (AF514410) | 13. *Pseuodchlorella signiensis* (KM116465) | 14. *Pseudochlorella pringsheimii* (FM958479) |
|---|---|---|---|---|---|---|---|---|---|---|---|---|---|---|
| *Chlamydomonas nivalis* (GU117577) 1. | 0 | | | | | | | | | | | | | |
| Chlainomonas sp. LP03 (MF803745) 2. | 40 | 0 | | | | | | | | | | | | |
| *Chloromonas nivalis* tetrae (KY499614.1) 3. | 25 | 21 | 0 | | | | | | | | | | | |
| *Chloromonas fukushimae* (AB906342) 4. | 26 | 24 | 5 | 0 | | | | | | | | | | |
| *Chloromonas tughillensis* (AB734116) 5. | 27 | 23 | 6 | 1 | 0 | | | | | | | | | |
| *Chloromonas nivalis* (GU117576) 6. | 25 | 21 | 0 | 5 | 6 | 0 | | | | | | | | |
| *Chloromonas polyptera* (JQ790556) 7. | 23 | 23 | 2 | 7 | 8 | 2 | 0 | | | | | | | |
| *Chloromonas hohamii* (AB906344) 8. | 27 | 23 | 6 | 1 | 0 | 6 | 8 | 0 | | | | | | |
| *Chloromonas brevispina* (AF517092) 9. | 27 | 23 | 6 | 1 | 0 | 6 | 8 | 0 | 0 | | | | | |
| *Chloromonas chenangoensis* (AB734113) 10. | 24 | 22 | 9 | 8 | 7 | 9 | 11 | 7 | 7 | 0 | | | | |
| *Raphidonema nivalis* (AJ306532) 11. | 21 | 37 | 23 | 22 | 23 | 23 | 25 | 23 | 23 | 20 | 0 | | | |
| *Raphidonema sempervirens* (AF514410) 12. | 21 | 37 | 23 | 22 | 23 | 23 | 25 | 23 | 23 | 20 | 0 | 0 | | |
| *Pseuodchlorella signiensis* (KM116465) 13. | 20 | 35 | 22 | 21 | 21 | 22 | 24 | 21 | 21 | 18 | 3 | 3 | 0 | |
| *Pseudochlorella pringsheimii* (FM958479) 14. | 20 | 35 | 22 | 21 | 21 | 22 | 24 | 21 | 21 | 18 | 3 | 3 | 0 | 0 |

**Supplementary figure S4.** **(A)** Number of base pair differences between GenBank sequence segments targeted by 18S primer pair Euk1181F-1624R used by Engstrom et al. (2020). **(B)** Number of base pair differences between GenBank sequence segments targeted by 18S primer pair 528F-706R used by Lutz et al. (2016) and Terashima et al. (2017).

# S5. Comparison of *rbcL* GenBank sequences

| | 1. *Chloromonas nivalis* (AB434272.1) | 2. *Chloromonas pseudoplatyrhyncha* (LC012752.1) | 3. *Chloromonas tughillensis* (LC012747.1) | 4. *Chloromonas brevispina* (AF517072.1) | 5. *Chloromonas fukushimae* (LC012738.1) | 6. *Chloromonas fukushimae* (LC012739.1) | 7. *Chloromonas miwae* (AB434267.1) | 8. *Chloromonas pichinchae* (EU030690.1) | 9. *Chloromonas remiasii* (LC360494.1) | 10. *Chlamydomonas radiata* (AJ001878.1) | 11. *Chloromonas asteroidea* (AB022225.1) | 12. *Chlainomonas rubra* (DQ885964.2) | 13. *Chlainomonas kolii* (DQ885962.1) | 14. *Chloromonas serbinowi* (AJ001879.1) | 15. *Chloromonas insignis* (AB022226.1) | 16. *Chloromonas reticulata* (AB022530.1) | 17. *Chloromonas kasaiae* (LC012751.1) | 18. *Chloromonas augustae* (AB504764.1) | 19. *Chloromonas chenangoensis* (EU030689.1) | 20. *Chloromonas chlorococcoides* (AB101508.1) |
|---|---|---|---|---|---|---|---|---|---|---|---|---|---|---|---|---|---|---|---|---|
| *Chloromonas nivalis* (AB434272.1) 1. | 0 | | | | | | | | | | | | | | | | | | | |
| *Chloromonas pseudoplatyrhyncha* (LC012752.1) 2. | 31 | 0 | | | | | | | | | | | | | | | | | | |
| *Chloromonas tughillensis* (LC012747.1) 3. | 30 | 31 | 0 | | | | | | | | | | | | | | | | | |
| *Chloromonas brevispina* (AF517072.1) 4. | 32 | 31 | 16 | 0 | | | | | | | | | | | | | | | | |
| *Chloromonas fukushimae* (LC012738.1) 5. | 34 | 28 | 13 | 16 | 0 | | | | | | | | | | | | | | | |
| *Chloromonas fukushimae* (LC012739.1) 6. | 34 | 28 | 13 | 16 | 0 | 0 | | | | | | | | | | | | | | |
| *Chloromonas miwae* (AB434267.1) 7. | 35 | 39 | 22 | 25 | 21 | 21 | 0 | | | | | | | | | | | | | |
| *Chloromonas pichinchae* (EU030690.1) 8. | 33 | 39 | 26 | 24 | 28 | 28 | 21 | 0 | | | | | | | | | | | | |
| *Chloromonas remiasii* (LC360494.1) 9. | 38 | 39 | 34 | 43 | 37 | 37 | 39 | 31 | 0 | | | | | | | | | | | |
| *Chlamydomonas radiata* (AJ001878.1) 10. | 42 | 32 | 36 | 36 | 36 | 36 | 42 | 46 | 46 | 0 | | | | | | | | | | |
| *Chloromonas asteroidea* (AB022225.1) 11. | 50 | 32 | 44 | 41 | 43 | 43 | 49 | 47 | 48 | 31 | 0 | | | | | | | | | |
| *Chlainomonas rubra* (DQ885964.2) 12. | 36 | 44 | 41 | 51 | 41 | 41 | 46 | 41 | 43 | 52 | 63 | 0 | | | | | | | | |
| *Chlainomonas kolii* (DQ885962.1) 13. | 35 | 46 | 42 | 50 | 46 | 46 | 45 | 38 | 38 | 44 | 54 | 15 | 0 | | | | | | | |
| *Chloromonas serbinowi* (AJ001879.1) 14. | 38 | 38 | 45 | 44 | 41 | 41 | 46 | 46 | 41 | 44 | 51 | 44 | 44 | 0 | | | | | | |
| *Chloromonas insignis* (AB022226.1) 15. | 47 | 43 | 43 | 48 | 44 | 44 | 45 | 45 | 46 | 43 | 45 | 45 | 47 | 42 | 0 | | | | | |
| *Chloromonas reticulata* (AB022530.1) 16. | 45 | 43 | 40 | 45 | 42 | 42 | 44 | 46 | 42 | 34 | 47 | 47 | 45 | 32 | 43 | 0 | | | | |
| *Chloromonas kasaiae* (LC012751.1) 17. | 36 | 35 | 36 | 37 | 34 | 34 | 41 | 39 | 41 | 37 | 46 | 44 | 45 | 36 | 31 | 36 | 0 | | | |
| *Chloromonas augustae* (AB504764.1) 18. | 43 | 40 | 43 | 41 | 39 | 39 | 50 | 46 | 53 | 39 | 46 | 55 | 59 | 44 | 31 | 44 | 36 | 0 | | |
| *Chloromonas chenangoensis* (EU030689.1) 19. | 35 | 46 | 38 | 39 | 38 | 38 | 42 | 40 | 30 | 46 | 49 | 42 | 43 | 46 | 46 | 46 | 45 | 54 | 0 | |
| *Chloromonas chlorococcoides* (AB101508.1) 20. | 62 | 57 | 56 | 59 | 56 | 56 | 53 | 58 | 53 | 62 | 64 | 70 | 69 | 65 | 63 | 59 | 55 | 60 | 67 | 0 |

**Supplementary figure S5.** Number of base pair differences between GenBank sequence segments targeted by *rbcL* primer pair rbcL369F-870R used by Engstrom et al. (2020).

## S6. *rbcL* and 18S primer sequences

| Name | Sequence (5'-3') |
| --- | --- |
| rbcL369F | GAACGTGACAAATTAAACAAA |
| rbcL870R | ACCWGAYADACGWAGAGCTT |
| Euk1181 (Wang et al. 2014) | TTAATTTGACTCAACRCGGG |
| Euk1624 (Wang et al. 2014) | CGGGCGGTGTGTACAAAGG |

Reference: Wang Y, Tian RM, Gao ZM, Bougouffa S, Qian P-Y. Optimal Eukaryotic 18S and Universal 16S/18S Ribosomal RNA Primers and Their Application in a Study of Symbiosis. PLOS ONE 2014; 9: e90053.

## S7. PCR conditions used for Illumina amplicon library prep

We constructed our amplicon library using a two-step PCR. In the first PCR we amplified template DNA using our primers attached to a universal adapter, and in the second PCR we re-amplified that product to attach a 6 bp index to the universal adapter at the 3' end. The first PCR total volume was 25 $\mu$L, consisting of 1 $\mu$L template, 12.5 $\mu$L Q5 high-fidelity 2X Master Mix (New England BioLabs), 1.25 $\mu$L each of forward and reverse primer, and 9 $\mu$L of ddH2O. The second PCR was the same except we reduced our reaction volume to 20 $\mu$L by using only 5 $\mu$L of ddH2O. The cycling conditions were the same for both primer pairs for the first PCR, with an initial denaturation at 98 °C for 30 s, followed by 30 cycles of 98 °C for 5 s, 58 °C for 10 s, and 72 °C for 25 s, with a final extension at 72 °C for 2 minutes. For the second indexing PCR we started with an initial denaturation at 98 °C for 30 s, then 10 cycles of 98 °C for 10 s, 65 °C for 30 s, and 72 °C for 30 s with a final denaturation of 72 °C for 5 min.

# S8. Phylogenetic tree of *rbcL* ASVs

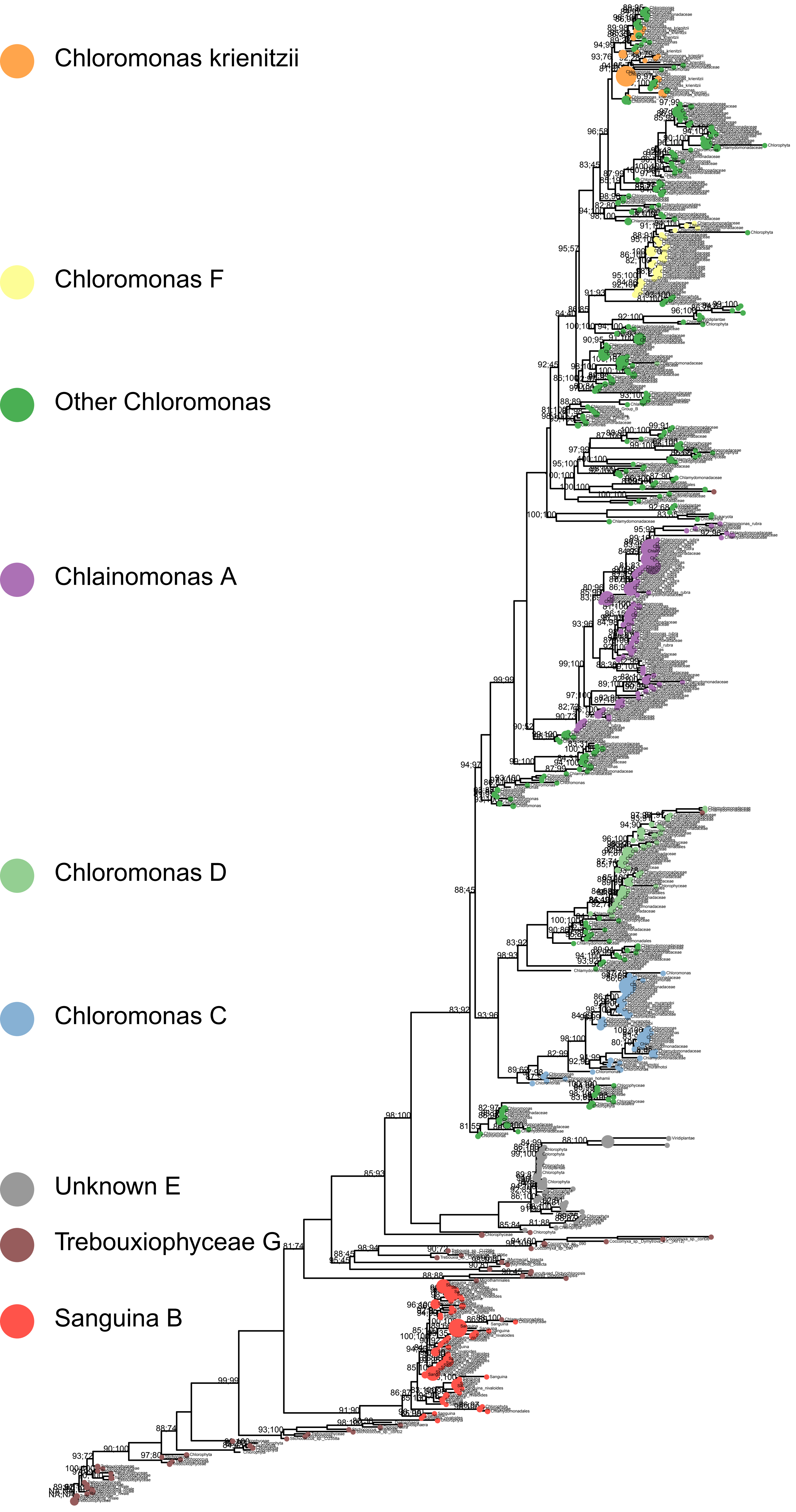


**Supplementary figure S8.** Maximum likelihood phylogenetic tree of our *rbcL* ASVs. Maximum likelihood phylogenetic tree of our *rbcL* ASVs (~400bp). Nodes annotated with bootstrap;bayesian scores, tips with best taxonomic annotation. Coloured circles on tips represent OTU, size proportional to total relative abundance.

# S9. 18S Chlorophyceae ASV clustering

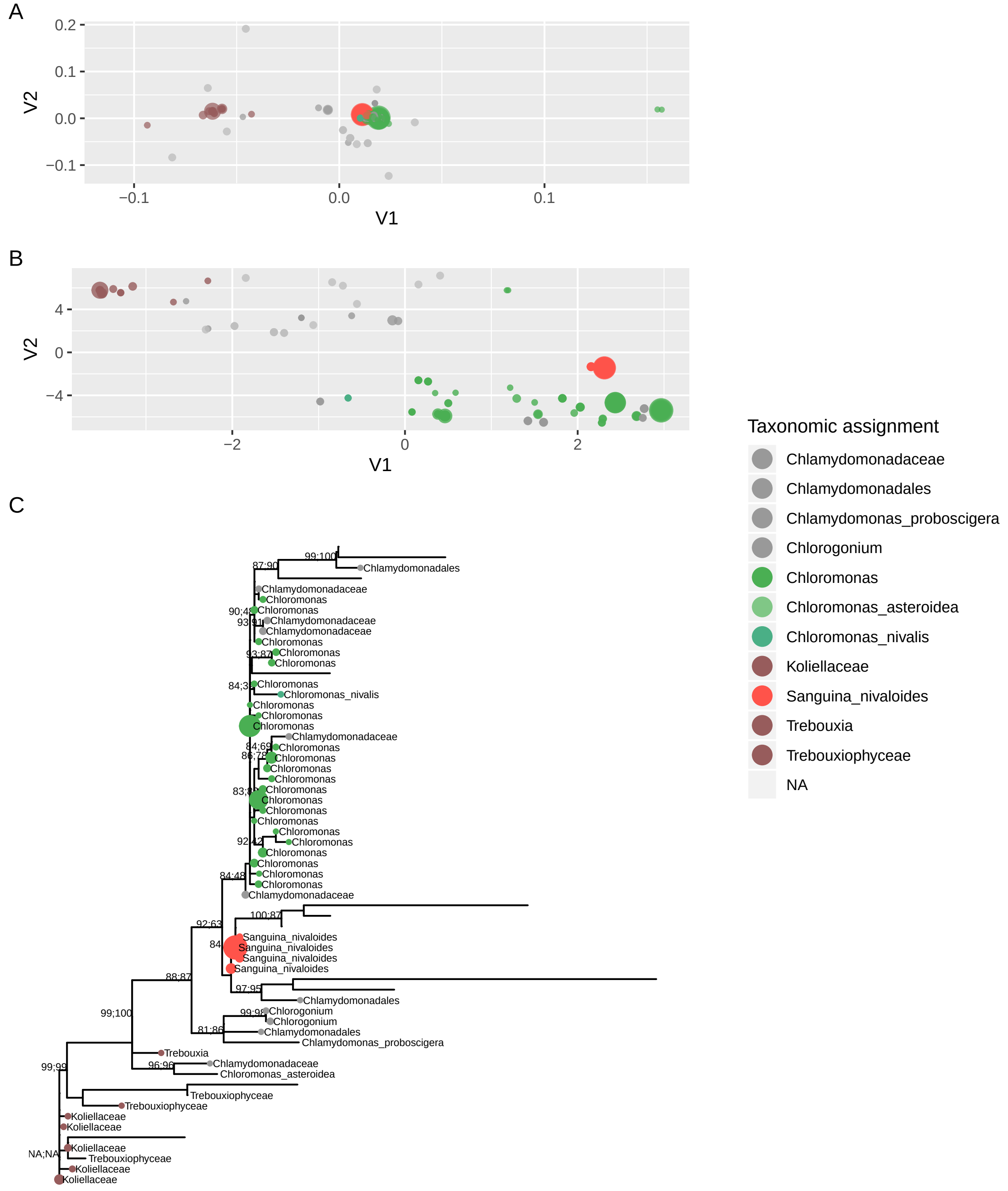


**Supplementary figure S9.** Genetic distance between 18S algae ASVs (the subset assigned to Chlorophyta by SILVA). Legend shows taxonomic assignment using custom GenBank snow algae database, size proportional to total relative abundance. ASVs**(A)** Multi-dimentional scaling (MDS). **(B)** t-SNE clustering of genetic distance between 18S, perplexity=20. **(C)** Maximum likelihood phylogenetic tree (IQTree).

# S10. Cell count relative abundance

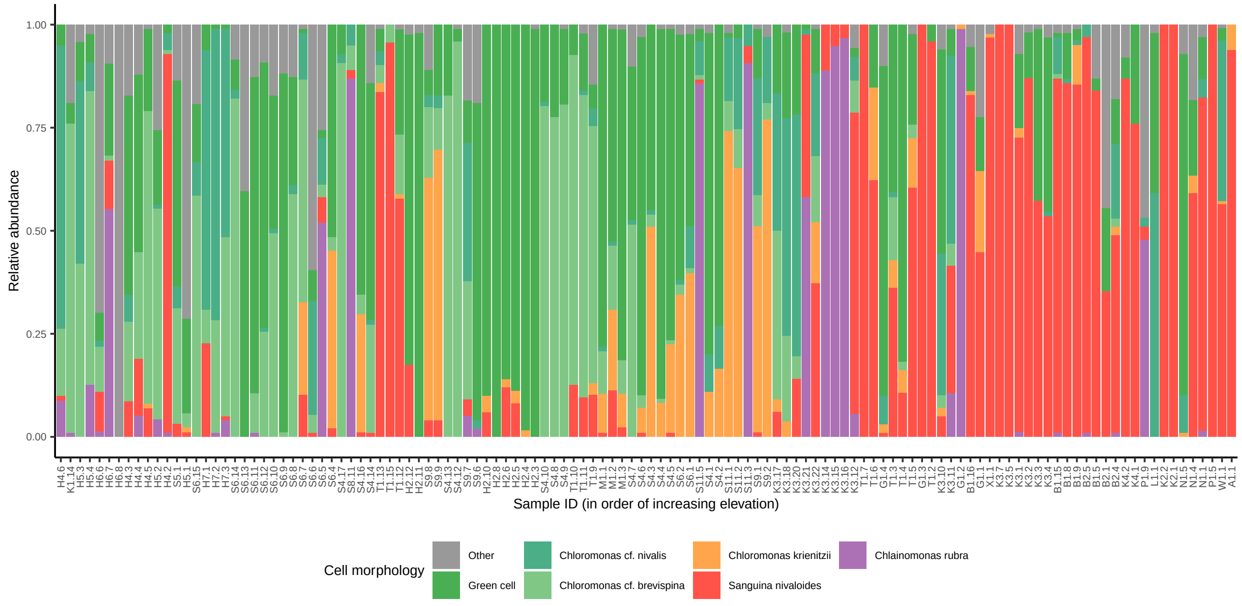


**Supplementary figure S10.** Cell count relative abundances of snow algae samples, with samples arranged on the x axis from lowest to highest elevation. Colors represent morphological species.

## S11. *rbcL* sample diversity by date and elevation

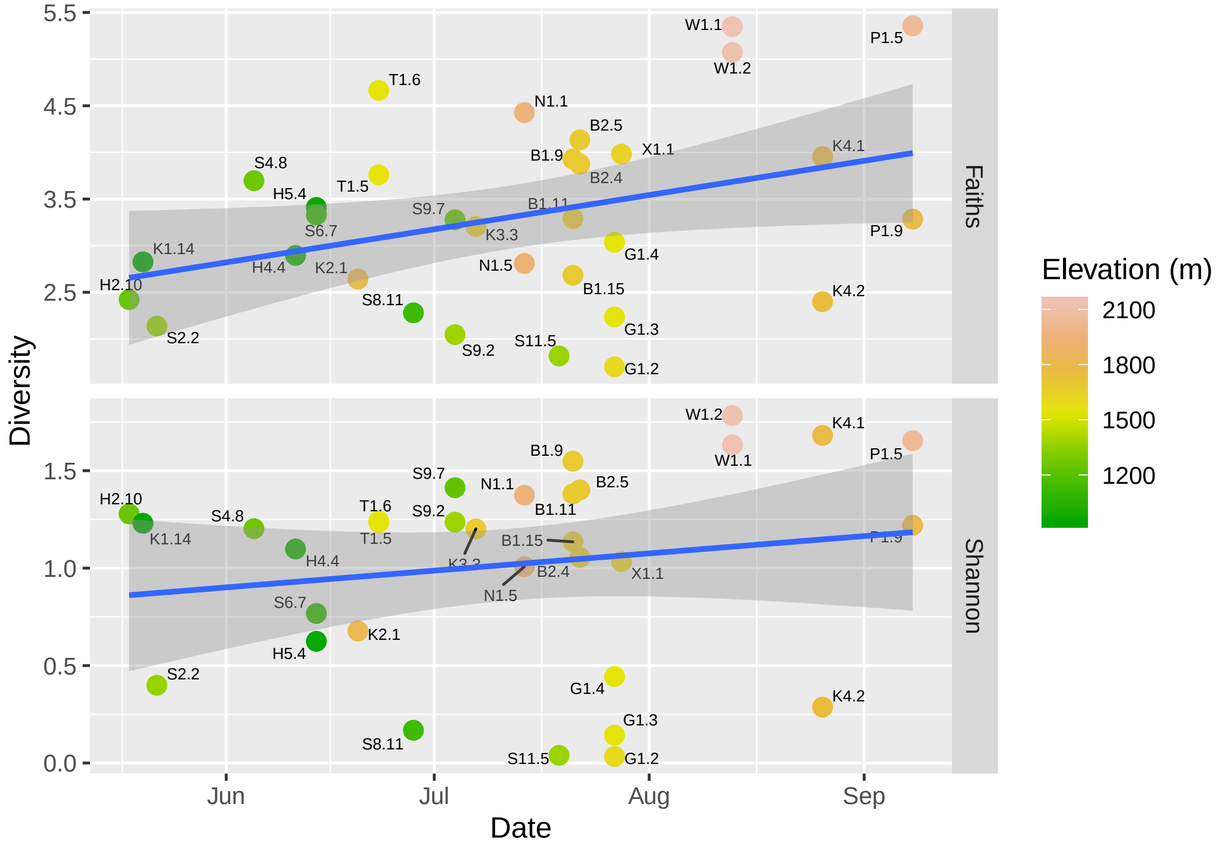


**Supplementary figure S11.** Faith's phylogenetic diversity (upper) and Shannon diversity (lower) of snow algae *rbcL* samples, plotted by date of sample collection. Samples colored by elevation and labelled by sample ID. Faith's Pearson's $r$=0.36, p=0.04, Shannon's Pearson's $r$=0.17, p=0.35.

## S12. Community comparison: same site, different dates & depths

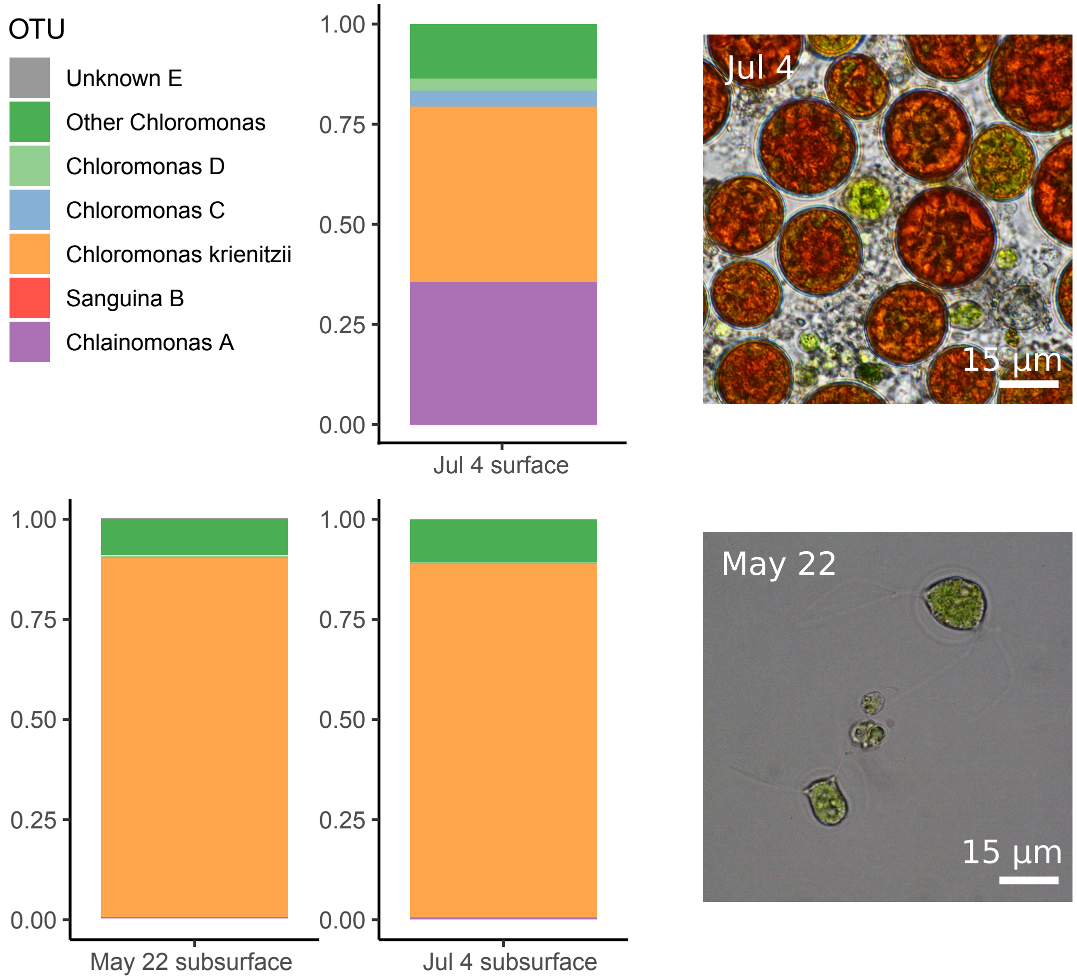


**Supplementary figure S12.** Temporal and depth comparison of a snow algae runnel. **(A)** *rbcL* relative abundance of OTUs from May 22 green subsurface snow (S2.2), and samples from July 4 (S9.1, S9.2) with orange snow at surface and green snow below. **(B)** Representative light photomicrographs of cells from S9.2 (top), S2.2 (bottom).
